# Molecular Interactions between NAFLD and Xenobiotic Metabolism

**DOI:** 10.3389/fgene.2013.00002

**Published:** 2013-01-22

**Authors:** Adviti Naik, Aleš Belič, Ulrich M. Zanger, Damjana Rozman

**Affiliations:** ^1^Faculty of Computer Sciences and Informatics, University of LjubljanaLjubljana, Slovenia; ^2^Faculty of Electrical Engineering, University of LjubljanaLjubljana, Slovenia; ^3^Dr. Margarete Fischer-Bosch Institute of Clinical PharmacologyStuttgart, Germany; ^4^University of TuebingenTuebingen, Germany; ^5^Centre for Functional Genomics and Bio-Chips, Institute of Biochemistry, Faculty of Medicine, University of LjubljanaLjubljana, Slovenia

**Keywords:** NAFLD, xenobiotic metabolism, nuclear receptors, phase I and II enzymes, transporters

## Abstract

Non-alcoholic fatty liver disease (NAFLD), the hepatic manifestation of the metabolic syndrome, is a complex multifactorial disease characterized by metabolic deregulations that include accumulation of lipids in the liver, lipotoxicity, and insulin resistance. The progression of NAFLD to non-alcoholic steatohepatitis and cirrhosis, and ultimately to carcinomas, is governed by interplay of pro-inflammatory pathways, oxidative stress, as well as fibrogenic and apoptotic cues. As the liver is the major organ of biotransformation, deregulations in hepatic signaling pathways have effects on both, xenobiotic and endobiotic metabolism. Several major nuclear receptors involved in the transcription and regulation of phase I and II drug metabolizing enzymes and transporters also have endobiotic ligands including several lipids. Hence, hepatic lipid accumulation in steatosis and NAFLD, which leads to deregulated activation patterns of nuclear receptors, may result in altered drug metabolism capacity in NAFLD patients. On the other hand, genetic and association studies have indicated that a malfunction in drug metabolism can affect the prevalence and severity of NAFLD. This review focuses on the complex interplay between NAFLD pathogenesis and drug metabolism. A better understanding of these relationships is a prerequisite for developing improved drug dosing algorithms for the pharmacotherapy of patients with different stages of NAFLD.

## Introduction

Non-alcoholic fatty liver disease (NAFLD) is the most common chronic liver disease in Western countries with a wide disease spectrum. It ranges from the hepatic accumulation of lipids known as steatosis, non-alcoholic steatohepatitis (NASH) wherein steatosis is accompanied by inflammation and can further progress to cirrhosis and hepatocellular carcinoma. NAFLD is the hepatic manifestation of the metabolic syndrome as it is frequently associated with obesity, insulin resistance, hyperglycemia, hypertension, and dyslipidemia (Anderson and Borlak, 2008; Lewis and Mohanty, 2010; Cohen et al., 2011).

The accumulation of hepatic triglycerides due to an imbalance between free fatty acids uptake, *de novo* lipogenesis, oxidation, esterification, and secretion, is a hallmark of hepatic steatosis (Donnelly et al., 2005). Initially the progression of NAFLD to NASH was described as a two-hit event, with the accumulation of hepatic triglycerides being the first hit, which in turn triggers the second hit – inflammation and oxidative stress (Crespo et al., 2001; Browning and Horton, 2004; Sunny et al., 2011; Wree et al., 2011). An extended hypothesis proposed a reduced capacity of hepatic regeneration and the detrimental effects of free fatty acid lipotoxicity as the third hit (Feldstein et al., 2004; Day, 2006).

Several other factors contribute to the induction of NAFLD and/or to its progression. These include compromised adipose tissue storage and function (Calvert et al., 2007), upregulation of inflammatory factors, and reactive oxygen species (ROS; Crespo et al., 2001; Browning and Horton, 2004; Wree et al., 2011), the interaction of the intestinal microbial populations with the host through the inflammasome (Henao-Mejia et al., 2012), downregulation of the endoplasmic reticulum stress and protein ubiquitination pathways (*HSPA5*, *USP25*), and gene expression changes in cell development, morphology, movement, death, and antigen presentation pathways (Gawrieh et al., 2010). Insulin resistance also plays a detrimental role in the pathogenesis of NAFLD. Polymorphisms that may potentially explain this effect were identified in the insulin receptor-substrate 1 (*IRS-1*; Gly172Arg; Dongiovanni et al., 2010).

As a multifactorial disorder, NAFLD is characterized by interactions between genetic and environmental factors, thus proving it difficult to understand its manifestations (Fon Tacer and Rozman, 2011; Lorbek and Rozman, 2012). Moreover, the scarcity of robust non-invasive diagnostic methods represents an obstacle in accurately determining the prevalence of NAFLD (Ratziu et al., 2011). By current estimations, NAFLD has a prevalence rate of 6–35% with a median of 20%, depending on the population studied and the method of assessment (Vernon et al., 2011; Chalasani et al., 2012). Ethnic differences in the prevalence of NAFLD also exist, with a lower frequency in African–Americans compared to Hispanic-Americans (Wagenknecht et al., 2009; Stepanova et al., 2010). It occurs in children (Roberts, 2007) and adults of all age groups, however conflicting observations have been made regarding the gender-specific risk of NAFLD (Bedogni et al., 2005; Chen et al., 2008b). The prevalence of NASH is much lower, affecting 2–5% of the population (Neuschwander-Tetri and Caldwell, 2003; Vernon et al., 2011), however, its frequency escalates with increasing age, body mass index (BMI), more severe forms of insulin resistance, hypertriglyceridemia, and poor liver function. Increased progression to NASH is observed in Hispanics, possibly due to the large-scale adaptation of western lifestyles (Browning et al., 2004). Obese individuals (BMI > 30 kg/m^2^) are at a higher risk of developing NAFLD, with a prevalence of 85–98% for NAFLD and >25% for NASH (Machado et al., 2006). Furthermore, diabetes mellitus (Type 2 Diabetes, T2D) is a major determinant of NAFLD with a 70% prevalence of NAFLD in some diabetic populations (Targher et al., 2006; Leite et al., 2009). However, NAFLD also occurs in approximately 18% of normal weight non-diabetic populations (de Alwis and Day, 2008).

Due to the lack of targeted drugs, NAFLD patients are usually treated by cholesterol-lowering statins, fibrates, or anti-diabetics such as thiozolidinediones, sulfonylureas, etc. (Rozman and Monostory, 2010). The controversy regarding the NAFLD patients’ benefits versus the potential harm due to liver toxicity is however, a matter of investigations and vivid debates.

## NAFLD and Drug Disposition

Liver is the major organ of endogenous and xenobiotic metabolism. In healthy livers, the metabolic processes are in homeostasis. A long-term disturbance of one or more metabolic pathways can provoke liver diseases. The intracellular accumulation of xeno- and endo-biotics is potentially toxic and is regulated at several levels including uptake, biotransformation, and elimination by drug metabolizing enzymes (DMEs). DMEs are classified as phase I, mainly cytochrome P450s (CYPs) that catalyze hydroxylation reactions, or phase II enzymes that are involved in conjugation reactions. Human phase I DME subfamilies CYP3A and CYP2C account for 50% of all hepatic CYPs and metabolize a large number of diverse drugs, e.g., lovastatin, tamoxifen, and R-warfarin. Phase II DMEs include UDP-glucuronosyltransferases (UGTs), sulfotransferases (SULTs), *N*-acetyltransferases, and several other transferases that transform compounds into more excretable forms. Transporters such as organic anion transporting polypeptide (OATP) and ATP-binding cassette (ABC)-transporters are responsible for the hepatocellular uptake and excretion of xenobiotics. The expression of phase I and II DMEs and transporters is regulated by a battery of nuclear receptors in a coordinated manner (Aleksunes and Klaassen, 2012).

Non-alcoholic fatty liver disease patients show differences in drug metabolism and its regulatory components, as summarized in Tables 1– 3. Compared to normal subjects, a pediatric NAFLD population exhibited altered glucuronidation of acetaminophen, a commonly used analgesic and antipyretic agent known to cause acute hepatic failure (Barshop et al., 2011). Although the pharmacokinetic profile of acetaminophen in both the normal and NAFLD subjects was unaltered, this study highlighted changes in the biotransformation of the drug and the possibility of compensation by other excretory pathways in the presence of NAFLD. Similarly, the metabolism of ezetimibe, an intestinal cholesterol-uptake blocker, is altered in NASH patients due to differential localization of ABCC2 and ABCB1 efflux transporters, hence, resulting in plasma retention of the active glucuronide metabolite of ezetimibe (Hardwick et al., 2012b). Studies have also indicated a reduced efficacy of certain treatments in NAFLD condition, such as the novel oral hypoglycemic sitagliptin, a dipeptidyl peptidase-4 (DPP-4) inhibitor. T2D patients with NAFLD have increased serum DPP-4 activity, an enzyme that inhibits incretins such as glucagon-like peptide 1 (GLP-1), and thus, reduced efficacy of sitagliptin (Firneisz et al., 2010; Iwasaki et al., 2012).

**Table 1:** **Nuclear receptors and transcription factors in interaction between NAFLD and drug metabolism**.

Nuclear receptor/transcription factor	Targets	Association with NAFLD
Pregnane X receptor (PXR)	*CYP2C9* *CYP3A4* *CYP2B6*	Activation in mice causes hepatic steatosis due to enhanced lipogenesis, decreased β-oxidation, and increased uptake of fatty acids via CD36 activation (Zhou et al., 2006b)
	*UGT1A1* *MDRP1 P-glycoprotein* *CD36*	A NAFLD population of European descent displayed strong association between rs2461823/A and rs7643645/G-containing haplotypes and disease severity irrespective of BMI and HOMA index (Sookoian et al., 2010)
Constitutive androstane receptor (CAR)	*CYC2C9*	*Car*+/+ mice fed MCD diet develop increased liver fibrosis (Yamazaki et al., 2007)
	*CYP2B6* *CYP3A4* *UGT1A1*	Activation results in the induction of aberrant hepatic DNL and insulin resistance via the expression of THRSP (Anderson et al., 2009; Rezen et al., 2009; Breuker et al., 2010)
		Lowers plasma concentration of HDL (Masson et al., 2008)
Farnesoid X receptor (FXR)	*CYP7A1* *BSEP*	Deficiency in a mouse model of hypercholesterolemia fed on a HFD results in features of NASH (Kong et al., 2009)
		FXR*1B (-1T) is associated with decreased FXR expression and functionality (Marzolini et al., 2007)
Liver X receptor (LXR)	*SREBP-1c* *PPAR*γ	Involved in lipid biosynthesis, cholesterol and bile acid homeostasis, and fatty acid uptake (Handschin and Meyer, 2005; Rezen et al., 2011)
	*chREBP* *CD36*	Plays a crucial function in glucose tolerance, insulin secretion, and adipocyte size (Efanov et al., 2004; Gerin et al., 2005)
	*ABC1*, *ABCG1*, *ABCG5*, *ABCG8*	rs17373080[G] polymorphism in *LXR*β associated with 20–30% lower risk of T2D prevalence but a higher risk of obesity (Dahlman et al., 2009; Solaas et al., 2010)
Peroxisome proliferator-activated receptor (PPAR)	*ACS* *CPT-I* *SULT 1C1*, *1C2*, *1E1*, *2A1*, *2A2*, *3A1* *UGT1A1*, *UGT1A3*, *UGT1A6*, *UGT2B4*	Fibrates are utilized to treat patients with elevated plasma triglycerides PPARα activates fatty acid oxidation and hepatic lipid hydrolysis and downregulates hepatic triglyceride secretion (Kersten et al., 1999; Pyper et al., 2010; Rakhshandehroo et al., 2010)
		*Ppar*α-deficient mice develop hepatic steatosis on a high-fat diet (HFD; Abdelmegeed et al., 2011)
		Potential protective role for the *Val227Ala* variant of *PPAR*α against obesity compared to wild-type variant (Chen et al., 2008a)
Nuclear factor erythroid 2-related factor 2 (Nrf2)	*GST* *HO-1*, *Nqo1*, *GCLC*	Null mice on MCD diet exhibit increased hepatic steatosis, inflammation, and oxidative stress (Chowdhry et al., 2010)
	*Mrp2/ABCC2*	Rats fed with an MCD diet display Nrf2-dependent upregulation of oxidative stress response (Lickteig et al., 2007)

Several drug metabolizing CYPs are downregulated in genetically modified (e.g., leptin deficient ob/ob, dysfunctional leptin receptor db/db mice) and diet-induced [e.g., high-fat diet, methionine- and choline-deficient (MCD) diet] NAFLD animal models and in patients with characteristics of the metabolic syndrome (Buechler and Weiss, 2011; Ghose et al., 2011). Genome wide association and candidate gene studies have identified single nucleotide polymorphisms (SNPs) in DMEs that associate with NAFLD prevalence, progression, or severity, highlighting the role of altered drug metabolism in NAFLD pathogenesis (Anstee et al., 2011; Lake et al., 2011). Thus, there is ample evidence for altered xenobiotic metabolism and efficacy in NAFLD patients.

A function of CYPs in hepatic lipid homeostasis is indicated by their broad role in microsomal oxidation, cholesterol biosynthesis, and their activation by lipids. Further proof of this role was observed from studies in the liver conditional CYPs reductase microsomal flavoprotein NADPH: CYP oxidoreductase (*Por*) null mice. POR is an essential redox partner of the microsomal CYPs. The liver conditional *Por* knockout mice display hepatomegaly, hepatic steatosis, and a reduced capacity of drug metabolism (Gu et al., 2003). One of the CYPs, CYP51, is an essential enzyme of cholesterol synthesis (Keber et al., 2011). Cholesterol and its metabolites are also known to contribute to lipotoxicity and inflammation-mediated progression of NAFLD to NASH (Caballero et al., 2009). The cholesterol synthesis pathway responds to feedback regulation by cholesterol, TNF-α stimulation (Tacer et al., 2007), and xenobiotics, such as statins (Rezen et al., 2008, 2009; Rozman and Monostory, 2010), thus emphasizing the hepatic crosstalk between endobiotic and xenobiotic metabolism and inflammation. Endobiotics and xenobiotics activate various nuclear receptors and thus influence the expression of genes involved in the various hepatic metabolic pathways (Gao and Xie, 2010). The interplay between metabolism of endobiotics and xenobiotics is a frequent cause of drug side effects that can now be explained at the molecular level (Hafner et al., 2011; Rezen, 2011).

### Nuclear receptors regulating DMEs

#### Pregnane X receptor

Pregnane X receptor (PXR, NR1I2) is a ligand-activated nuclear receptor that upon activation forms a heterodimer with retinoid X receptor (RXR) and regulates the expression of a wide range of DMEs (Tolson and Wang, 2010). Apart from being activated by xenobiotics, it also responds to endobiotics including bile acids (Xie et al., 2001) and steroid hormones (di Masi et al., 2009). In mice, PXR activation results in hepatic steatosis due to enhanced sterol regulatory element-binding protein-1c (SREBP-1c)-independent lipogenesis, decreased β-oxidation, and increased uptake of fatty acids via fatty acid translocase (FAT/CD36) activation (Zhou et al., 2006b). The activation of CD36 by PXR in mice occurs directly or *via* the activation of peroxisome proliferator-activated receptor gamma (PPARγ; Tontonoz et al., 1998; Zhou et al., 2006b). PXR also plays a role in glucose metabolism (Gao and Xie, 2012). It inhibits gluconeogenesis by inactivating hepatocyte nuclear factor-4 (HNF-4) and forkhead box protein O1 (FOXO1), both of which are positive regulators of gluconeogenic genes (Bhalla et al., 2004; Kodama et al., 2004). Genetic association studies in a European NAFLD population indicated an association of the *PXR* rs2461823[A] and rs7643645[G]-containing haplotypes and disease severity, irrespective of BMI and homeostatic model assessment (HOMA) index (Sookoian et al., 2010). Although PXR activation increases steatosis, PXR-dependent counteraction of inflammation by inhibition of nuclear factor kappa-B (NFκB) has also been documented in human and mouse samples (Zhou et al., 2006a). In support of the previous statement, rats treated with a PXR activator pregnenolone-16α-carbonitrile (PCN) displayed reduced hepatic fibrosis and necrosis in response to a fibrogenesis-inducing agent carbon tetrachloride (CCl_4_; Marek et al., 2005). It is thus plausible that *PXR* polymorphisms associated with NAFLD may affect disease severity by lowering PXR activity, thus resulting in increased susceptibility to NASH. Another link between PXR and NAFLD is through the modulation of DMEs. PXR induces the expression of CYP2C9 (Gerbal-Chaloin et al., 2002), which metabolizes the anti-diabetic drug rosiglitazone known to reduce liver fat. Since rosiglitazone is used in NAFLD patients with hyperglycemia and IR (Ratziu et al., 2008), patients with *PXR* gene variants may theoretically suffer from aberrant rosiglitazone metabolism. *PXR* is thus a potential pharmacogenetic marker for thiazolidinedione treatments. Furthermore, PXR is a strong modulator of CYP3A4, the major phase I DME in humans. Several SNPs that affect the expression of CYP3A4 reside within the PXR coding, non-coding, and promoter regions (Zhang et al., 2008). Since many NAFLD patients are treated with drugs metabolized by CYP3A4, further pharmacogenetic evaluation of patients with these *PXR* variants is required (Table 1).

#### Constitutive androstane receptor

Constitutive androstane receptor (CAR, NR1I3) is also a key regulator of xenobiotic and endobiotic metabolism (Moore et al., 2000; Handschin and Meyer, 2005). Upon ligand activation, CAR is translocated to the nucleus where it binds to DNA elements of DME genes such as *CYP2B6*, *CYP3A4 CYP2Cs*, and others (Sueyoshi and Negishi, 2001; Gerbal-Chaloin et al., 2002; Faucette et al., 2006; Chen and Goldstein, 2009), as well as phase II enzymes involved in glucuronidation (Sugatani et al., 2005), sulfation, and drug transport (Tolson and Wang, 2010). Several studies also indicate a role of CAR in energy homeostasis (Wada et al., 2009). Hence, the activation of CAR for regulation of energy homeostasis may affect drug metabolism (Hafner et al., 2011). SREBP-1c, which is upregulated in hepatic steatosis, inhibits CAR and thus, may further contribute to aberrant xenobiotic and endobiotic metabolism (Roth et al., 2008). *Car*+/+ mice fed with MCD diet, known to induce NASH (Rinella et al., 2008), developed enhanced liver fibrosis due to lipid peroxidation, inducible nitric oxide synthase (iNOS), and increased CYP induction compared to *Car*−/− mice (Yamazaki et al., 2007). However, no difference in hepatic lipid accumulation was observed between *Car*+/+ and *Car*−/− mice, indicating that CAR may be involved in later stages of NAFLD progression and hepatocarcinogenesis (Takizawa et al., 2011). Furthermore, observations in *Car*−/− mice and human hepatocytes highlight the role of CAR activation in the induction of aberrant hepatic *de novo* lipogenesis and insulin resistance by enhancing the expression of thyroid hormone-responsive spot 14 protein (*THRSP*; Anderson et al., 2009; Breuker et al., 2010). Activation of CAR in mice with 1,4-Bis [2-(3,5-dichloropyridyloxy)] benzene (TCPOBOP) increased serum triglycerides and hepatic fatty acid synthesis and repressed adaptation to hyperlipidemia, which is expected to favor the development of NAFLD (Rezen et al., 2009). Contradictory observations arise from other mouse models, where CAR has been linked to improved fatty liver due to decreased lipogenesis, increased β-oxidation, improved glucose tolerance, and insulin sensitivity (Dong et al., 2009; Gao et al., 2009). In mice, CAR also regulates cholesterol and bile acid metabolism by lowering plasma high-density lipoprotein (HDL) and reverse cholesterol transport, possibly *via* downregulation of apolipoprotein A1 (ApoA1; Masson et al., 2008). *CAR* polymorphisms have not yet been linked to metabolic diseases; however, its role in glucose and lipid metabolism and its functional redundancy with PXR highlights that the *CAR* gene is an important candidate for NAFLD association studies (Rezen et al., 2009).

#### FXR and LXR

Farnesoid X receptor (FXR) and Liver X receptor (LXR) are not major regulators of xenobiotic metabolism, but they play an important role in the metabolism of cholesterol and bile acids (Rezen et al., 2011). FXR/NR1H4 is the predominant regulator of bile acid synthesis and secretion, thereby lowering hepatic cholesterol levels. The first and rate-limiting step of bile acid synthesis from cholesterol is catalyzed by cholesterol 7α-hydroxylase (CYP7A1). The activation of FXR in primary human and mouse hepatocytes results in decreased transcription of *CYP7A1* due to an indirect negative feedback mechanism (Goodwin et al., 2000; Holt et al., 2003). Furthermore, activated FXR upregulates the expression of CYP3A4, which hydroxylates some common bile acids into more soluble forms (Gnerre et al., 2004), as well as the bile salt export pump (BSEP; Ananthanarayanan et al., 2001; Plass et al., 2002; Song et al., 2008). Deficiency of FXR in an *Ldlr*−/− mouse model of hypercholesterolemia on high-fat diet results in features of NASH, such as macrosteatosis, hepatocyte ballooning, and inflammation (Kong et al., 2009). A common SNP, FXR*1B (-1T), has been identified in the sequence flanking the start codon of *FXR* in European, African, Chinese, and Hispanic-American populations. It associates with decreased FXR expression and functionality, which may contribute to the pathogenesis of metabolic disorders (Marzolini et al., 2007). However, association of this *FXR* polymorphism with NAFLD in humans has not yet been identified.

Liver X receptor plays important roles in lipid biosynthesis as well as cholesterol and bile acid homeostasis (Handschin and Meyer, 2005; Rezen et al., 2011). Typical activators of LXR are oxysterols such as 22(R)-hydroxycholesterol (22(R)-HC), 24(S), 25-epoxycholesterol, and 25-hydroxycholesterol (Jakobsson et al., 2012). LXR activation not only increases cholesterol catabolism but also hepatic lipogenesis through activation of SREBP-1c, PPARγ, or carbohydrate response element-binding protein (chREBP; Lee et al., 2008). LXR and PXR share many target genes (Boergesen et al., 2012) and both regulate the uptake of fatty acids into hepatocytes *via* expression of FAT *CD36* (Zhou et al., 2006b). Although LXR activates *Cyp7a1* expression during bile acid synthesis in rodents, it does not have an effect on human *CYP7A1* expression (Goodwin et al., 2003). In humans, the LXRα isoform is mainly expressed in tissues involved in lipid metabolism, where it regulates the expression of cholesterol transporter genes, such as the ABC transporters *ABC1* (Schwartz et al., 2000), *ABCG1* (Sabol et al., 2005), *ABCG5* (Repa et al., 2002), and *ABCG8* (Repa et al., 2002). LXRβ, the ubiquitously expressed isoform and the only isoform present in pancreatic β-cells, does not play a role in cholesterol homeostasis (Alberti et al., 2001) but has a crucial role in glucose tolerance, insulin secretion, and adipocyte size (Efanov et al., 2004; Gerin et al., 2005). The rs17373080[G] polymorphism in *LXR*β associates with a 20–30% lower risk of T2D but with a higher risk of obesity, as observed in two independent studies (Dahlman et al., 2009; Solaas et al., 2010). This is in accordance with data on *Lxr*β-null mice that display a lean phenotype with glucose intolerance (Gerin et al., 2005). According to the best of our knowledge, *LXR* polymorphisms have not yet been linked to NAFLD.

#### Peroxisome proliferator-activated receptors

Peroxisome proliferator-activated receptors (PPARs) are transcription factors that are activated by endogenous ligands, such as fatty acids, and synthetic ligands, such as the hypolipidaemic fibrates and the insulin-sensitizing thiozolidinediones. Like PXR and CAR, they form heterodimers with RXR and transactivate numerous target genes with vital roles in metabolism by binding to PPAR response elements (PPRE; Nielsen et al., 2008; van der Meer et al., 2010). The identified subtypes *PPAR*α, *PPAR*γ, and *PPAR*β*/*δ have different tissue-specificities and functions (Kallwitz et al., 2008). Whilst *PPAR*γ is highly expressed in adipose tissue and functions in adipocyte differentiation, *PPAR*α functions as a major regulator of lipid and glucose metabolism in the liver. *PPAR*β*/*δ is ubiquitously expressed with a wide array of functions. *PPAR*α-agonists, the fibrates, are utilized to treat patients with elevated plasma triglycerides (Sirtori and Franceschini, 1988) due to the ability of PPARα to activate fatty acid oxidation and hepatic lipid hydrolysis by regulating acyl CoA synthetase (*Acs*), carnitine palmitoyl transferase I (*Cpt-I*), mitochondrial β-oxidation enzymes, and hepatic lipases in addition to downregulating *apoCIII* and decreasing hepatic triglyceride secretion (Kersten et al., 1999; Pyper et al., 2010; Rakhshandehroo et al., 2010). Accordingly, *Ppar*α-deficient mice develop hepatic steatosis on a high-fat diet (Abdelmegeed et al., 2011).

A case-control study of NAFLD patients highlighted a potentially protective role for the *Val227Ala* variant of *PPAR*α against obesity compared to subjects with the wild-type receptor (Chen et al., 2008a). The adipocyte differentiation regulator, PPARγ, plays an important role in lipid homeostasis and insulin sensitivity by enhancing fatty acid and insulin-dependent glucose uptake in adipose tissue (Kallwitz et al., 2008). Moreover, novel *PPAR*γ agonists acting mainly on adipose *PPAR*γ prevent formation of steatotic livers in mice by improving insulin resistance, upregulating adiponectin, and downregulating leptin expression and secretion (Zheng et al., 2011). *PPAR*γ is expressed at low levels in the liver but is upregulated in rodent fatty livers, contributing to hepatic triglyceride accumulation with a protective effect to dyslipidemia and insulin resistance in other tissues (Gavrilova et al., 2003). Upregulation of hepatic *PPAR*γ is also observed in obese NAFLD patients (Pettinelli and Videla, 2011).

PPARα affects the expression of several phase II enzymes such as SULTs and UGT (Runge-Morris and Kocarek, 2009) as well as of *CYP3A4* and several other CYPs in humans (Rakhshandehroo et al., 2009; Klein et al., 2012). Contrasting observations have been made regarding the regulation of DMEs by PPARα in human versus mouse. While treatment of human primary hepatocytes with the selective PPARα agonist WY14 643 resulted in the activation of several drug metabolizing CYPs including *CYP3A4*, *CYP2B6*, *CYP2C8*, and *CYP1A2*, none of the mouse gene orthologs were regulated (Rakhshandehroo et al., 2009). Downregulation of *CYP3A4* in the presence of *PPAR*α variants that result in decreased hepatic PPARα protein levels was also found by genetic association analysis and confirmed in a human atorvastatin volunteer study (Klein et al., 2012). Moreover, activation of PPARα by agonists downregulates the expression of representatives genes of the *Sult 1*, 2, 3, and *5* families, specifically in female rats (Alnouti and Klaassen, 2008). However, in human hepatocytes activation of PPARα resulted in the upregulation of *SULT2A1* via a functional PPRE, further emphasizing species and gender differences in the functionality of PPARα (Fang et al., 2005). Protein and mRNA levels of UGT1A1 increased upon PPARα activation in rat and human hepatocytes (Jemnitz et al., 2000; Richert et al., 2003). Additionally, *UGT1A3*, *UGT1A4*, and *UGT1A6* are upregulated in human hepatocytes and transgenic mice carrying the human *UGT1* locus (Senekeo-Effenberger et al., 2007). *UGT2B4* is also enhanced after treatment of human hepatocytes with PPARα agonists (Barbier et al., 2003). Functional PPREs have been identified in the 5′-flanking regions of *UGT1A1*, *UGT1A3*, *UGT1A6*, and *UGT2B4* genes, thus providing evidence that these genes are direct targets of PPAR (Barbier et al., 2003; Senekeo-Effenberger et al., 2007). The widespread use of drugs metabolized by SULTs and UGTs such as hormonal contraceptives, acetaminophen, β_2_-adrenergic agonists, anti-depressants, and non-steroidal anti-inflammatory drugs highlights the implications of altered PPARα activation on xenobiotic metabolism in NAFLD patients.

#### Nuclear factor erythroid 2-related factor 2

Nuclear Factor Erythroid 2-related factor 2 (NRF2) is a transcription factor that responds to oxidative/electrophilic stimuli by releasing from its repressor Kelch-like ECH associating protein 1 (Keap1) in the cytosol, translocating to the nucleus, binding to antioxidant response elements (AREs) upstream of numerous phase II DME genes, and genes involved in redox balance and oxidative stress response [e.g., heme oxygenase-1 (*HO-1*), NAD(P)H:quinone oxidoreductase-1 (*NQO1*)] and activating their transcription (Wu et al., 2012). NRF2 also regulates the glutathione synthesis enzyme, glutamate cysteine ligase catalytic (*GCLC*). The expression of a canalicular biliary efflux transporter, multidrug resistance protein 2 (*MRP2/ABCC2*) and sinusoidal transporters, *MRP3* and *MRP4* is also regulated by NRF2 in mouse liver and HepG2 cells in response to oxidative stress and xenobiotics, thus providing further evidence that phase II enzymes and efflux transporters are regulated simultaneously (Vollrath et al., 2006; Aleksunes et al., 2008). MRP2 is involved in the excretion of reduced and oxidized glutathione and hence plays an important role in detoxification and against oxidative stress. *Nrf2*-null mice on MCD diet exhibit increased hepatic steatosis accompanied by inflammation and oxidative stress (Chowdhry et al., 2010). Similarly, in rats on MCD diet, the NRF2-dependent genes involved in the oxidative stress response were upregulated (Lickteig et al., 2007). Thus, NRF2 appears to have a crucial role in the pathogenesis of NAFLD.

Thus, it is evident that as many of the lipids that accumulate in obesity and steatosis, such as fatty acids, cholesterol, or bile acids, are endogenous ligands of nuclear receptors, their deregulation may not only exacerbate the deregulated metabolic processes in NAFLD patients but also result in deregulated xenobiotic metabolism.

### Phase I DMEs

#### CYP3A

The CYP3A sub-family of DMEs plays a predominant role in the metabolism of statins. Statins, in monotherapy and in combination with other lipid-lowering drugs or antioxidants, are beneficial in NAFLD patients by improving dyslipidemia (Athyros et al., 2011; Fon Tacer and Rozman, 2011). The inter-individual variability in the response to statins varies in NAFLD patients based on their risk for cardiovascular diseases (Maroni et al., 2011). Moreover, the CYP3A4 drug metabolizing activity is also a factor influencing inter-individual variability and hence, is relevant to NAFLD patients undergoing statin therapy. The level of CYP3A protein correlates negatively with the severity of steatosis in humans (Kolwankar et al., 2007). No changes were found in the CYP3A4 mRNA level in human fatty liver samples at various stages of NAFLD progression, however a trend of decreasing activity and protein levels was observed (Fisher et al., 2009b). In another study, CYP3A4 activity significantly decreased in macrosteatotic fatty livers and cultured human hepatocytes treated with fatty acids (Donato et al., 2006, 2007). *CYP3A4* also displays sexual dimorphism with approximately twofold elevated expression in premenopausal women (Wolbold et al., 2003), who display a more favorable lipid profile compared to men (Williams, 2004). An intron 6 polymorphism in *CYP3A4* (rs35599367[T]) results in decreased expression and activity of CYP3A4, with carriers of the T allele requiring significantly lower doses of statins (Elens et al., 2011; Wang et al., 2011). In accordance with the reduced CYP3A4 expression in NAFLD, studies to determine the association of the rs35599367 *CYP3A4* polymorphism in NAFLD cohorts will enable the elucidation of statin dose selection in these patients. Moreover, genetic variants in other factors implicated in NAFLD, endobiotic, and xenobiotic metabolism such as *PXR*, *PPAR*α, and *POR* have also been associated with altered CYP3A4 expression and activity (Zhang et al., 2008; Gomes et al., 2009; Klein et al., 2012). These studies emphasize the high level of variability in responses to statin treatments and may provide a basis for dose selection in NAFLD patients based on CYP3A4 status (Table 2).

**Table 2:** **Phase I drug metabolizing enzymes implicated in the pathogenesis of NAFLD**.

Phase 1 DME	Drugs metabolized/transported	Association with NAFLD
CYC2C9	Roziglitazone (anti-diabetic) Sulphanylureas (anti-diabetic) Warfarin (anti-coagulant) Tamoxifen (selective estrogen receptor modulator)	Loss-of-function variants associated with increased response to sulfonylurea drugs, a NAFLD treatment, and an increased glycemic response in the treatment of T2D patients (Zhou et al., 2010) mRNA and enzyme activity increases with NAFLD progression (Fisher et al., 2009b)
CYP3A4	Atorvastatin (statin) Simvastatin (statin)	Expression and activity affected by SNPs in the *PXR* coding, non-coding, and promoter regions, in *PPAR*α and *POR* (Zhang et al., 2008; Gomes et al., 2009; Klein et al., 2012)
	Lovastatin (statin) Fibrates (anti-dyslipidemia) Nateglinide (anti-diabetic)	Intron 6 SNP rs35599367[T] in *CYP3A4* results in decreased expression and activity of CYP3A4 and carriers of the T allele require significantly lower doses of statins to treat dyslipidemia (Elens et al., 2011; Wang et al., 2011)
	Docetaxel (anti-cancer)	CYP3A activity shows a negative correlation with the severity of steatosis (Kolwankar et al., 2007)
		Displays sexual dimorphism with elevated expression in premenopausal women with a more favorable lipid profile, compared to men (Wolbold et al., 2003)
CYP2E1	Propranolol (beta-blocker) Paracetamol (analgesic)	Catalyzes fatty acid oxidation in hepatic microsomal compartments and is implicated in NASH development (Williams, 2004)
		NAFLD and NASH patients and animal models display enhanced expression of CYP2E1 and lipid peroxidation (Robertson et al., 2001), with increased localization to areas in the liver with oxidative stress injuries, leptinemia, reduced adiponectin levels and insulin resistance in NAFLD (Weltman et al., 1996, 1998)
		Contrasting observations indicated decreased *CYP2E1* mRNA and protein levels and no changes in its activity at progressive stages of NAFLD (Aubert et al., 2011)
		*Cyp2e1*-null mice that still displayed lipid peroxidation had increased expression of *Cyp4a10* and *Cyp4a14* genes (Fisher et al., 2009b; Mitsuyoshi et al., 2009)
CYP4A	Fatty acid derivatives	Enhanced activity results in increased production of ROS, thus contributing to steatohepatitis
		In contrast, *Ppar*α-null mice on an MCD diet are more prone to developing NASH in the absence of *Cyp4a* induction (Leclercq et al., 2000; Hardwick et al., 2009)

#### CYP2C9

CYP2C9 is the most abundant CYP of the CYP2C sub-family in human liver microsomes, accounting for the metabolism of a large number of clinically important drugs, especially some with a narrow therapeutic index, such as warfarin. The expression of CYP2C9 is coordinated by nuclear receptors such as CAR and PXR in association with nuclear factors and coactivators such as hepatocyte nuclear factor-4 alpha (HNF-4α) and PPARγ coactivator-1 alpha (PGC-1α), which is also involved in energy homeostasis (Chen and Goldstein, 2009). CYP2C9 has been closely associated with adverse drug reactions. Its mRNA and enzyme activity increase with NAFLD progression, hypoxia, and at later stages of NASH in humans (Fisher et al., 2009b). Previous observations linking CYP2C9 with arachidonic acid metabolism and vasocontraction in hypoxic conditions (Pokreisz et al., 2006) may possibly provide an explanation for elevated CYP2C9 in progressive NAFLD. Approximately 50 variants have been identified in the *CYP2C9* gene to date, with the CYP2C9*2 and CYP2C9*3 loss-of-function alleles as the most important. Heterozygotes and homozygotes for these polymorphisms are common in Caucasians, with frequencies of approximately 10–17 (CYP2C9*2) and 7% (CYP2C9*3). Both polymorphic alleles were associated with increased response to anti-diabetic sulfonylurea drugs and an increased glycemic response in T2D patients (Zhou et al., 2010). As NAFLD patients are treated with sulfonylureas, genotyping is clinically relevant. Further studies are needed to identify the association of the CYP2C9*2 and CYP2C9*3 variants with adverse drug reactions such as hypoglycemia and weight gain resulting from sulfonylurea treatment.

#### CYP2E1

CYP2E1, a fatty acid (Ω-1)-hydroxylase, catalyzes the oxidation of many low molecular weight molecules, including ethanol and acetone, a product of fatty acid oxidation. An important catalytic feature of CYP2E1 is the generation of ROS such as superoxide anion radical and hydrogen peroxide as a result of uncoupling of oxygen consumption with NADPH oxidation and as a by-product of lipid peroxidation (Robertson et al., 2001; Caro and Cederbaum, 2004). It is also involved in the biotransformation of xenobiotics such as acetaminophen, resulting in the generation of toxic reactive metabolites (Aubert et al., 2011). NAFLD and NASH patients and the MCD diet-fed rat model of NASH display enhanced expression of CYP2E1, which is in contrast to all other drug metabolizing CYPs, and elevated lipid peroxidation (Weltman et al., 1996, 1998; Videla et al., 2004) with increased localization to hepatic areas with oxidative stress injuries. Obese females with steatosis and NASH display elevated CYP2E1 protein levels and a positive correlation between the c2 allele of *Rsa1/Pst1* polymorphisms in *CYP2E1* and liver injury (Varela et al., 2008). Mice with silenced diacylglycerol acyltransferase 2 (*Dgat2*) on MCD diet display elevated *Cyp2e1* expression that correlates with increased lipid peroxidation and oxidative damage, thus highlighting the role of CYP2E1 in the progression to NASH in response to increased hepatic free fatty acids (Yamaguchi et al., 2007). An upregulation in *CYP2E1* has also been associated with leptinemia, reduced adiponectin levels, and insulin resistance in NAFLD (Aubert et al., 2011). This phenomenon is reversed in patients who have undergone bariatric surgery with resulting decreases in weight and hepatic steatosis (Bell et al., 2010). With the robust cellular protection mechanisms intact, increases in pro-oxidant molecules and CYP2E1 are counteracted by increased levels of glutathione (GSH). However, most NASH rodent models display lower GSH, indicating defects in the oxidative stress response pathways in progressive NAFLD. Nitrosylation of antioxidant enzymes superoxide dismutase (SOD) and catalase (CAT) is crucial in a *Cyp2e1*-overexpressing mouse model of NAFLD (Kathirvel et al., 2010) because increased levels of iNOS generates reactive nitrogen species (RNS), which nitrosylate antioxidant enzymes and decrease their activity. Thus, *CYP2E1* polymorphisms that associate with the progression of NAFLD to NASH may possibly trigger the combined detrimental effects of both ROS and RNS, which in combination with toxic metabolites from xenobiotic biotransformation may result in further aggravated liver injury in NAFLD patients.

Observations in a NAFLD pediatric population have indicated a direct correlation between lipid peroxidation and disease severity irrespective of CYP2E1 levels; however, the small sample size of this study and the possibility of alternative mechanisms of lipid peroxidation in early onset hepatic steatosis cannot be excluded (Bell et al., 2011). Other groups have also previously indicated decreased CYP2E1 mRNA and protein levels and no changes in CYP2E1 activity at progressive stages of NAFLD (Fisher et al., 2009b; Mitsuyoshi et al., 2009). Thus, no conclusive role of CYP2E1 in NAFLD can be described. Interestingly, *CYP4A* genes seem to compensate for microsomal lipid oxidation in the absence of CYP2E1 as observed in *Cyp2e1*-null mice that display lipid peroxidation and increased expression of *Cyp4a10* and *Cyp4a14* (Leclercq et al., 2000; Hardwick et al., 2009). This observation may possibly explain the absence of changes in CYP2E1 activity in some NAFLD populations.

#### CYP4A

CYP4A enzymes ω-hydroxylate fatty acids into dicarboxylic acids that are preferentially oxidized by peroxisomes. Genes of the *CYP4A* sub-family are induced by PPARα-agonists and in conditions of fasting. The enhanced activity of CYP4A results in increased production of ROS, thus contributing to steatohepatitis. In contrast, PPARα agonists prevent NASH by increasing β-oxidation. Moreover, *Ppar*α-null mice on MCD diet are more prone to developing NASH in the absence of *Cyp4a* induction (Ip et al., 2003). These observations suggest that the anti-steatotic effects of PPARα may be more potent than its activation of *CYP4A* genes, hence overriding the ROS-generating effects of CYP4A. In the absence of PPARα alternative oxidative stress mechanisms may act as causal factors.

### Phase II DMEs

Phase II DMEs are conjugative, detoxification enzymes that transform substrates into more excretable inactive forms or on the other hand may also be involved in bioactivation. Glutathione-*S*-transferases (GSTs) are present as different isoforms Alpha, Mu, and Pi and conjugate electrophilic compounds with reduced GSH (Hayes et al., 2005). While a GST A and P are upregulated with disease progression in the livers of NAFLD patients, GST M is significantly downregulated, thus highlighting the differential regulation of GST isoforms in NAFLD progression; however, the overall GST activity was decreased in these samples (Hardwick et al., 2010). GSTM2, GSTM4, and GSTM5 mRNA levels are expressed at lower levels in patients with steatosis and NASH (Younossi et al., 2005). GSTs play a significant role in controlling oxidative stress by conjugating harmful by-products of oxidative stress with GSH (Hayes et al., 2005). Decreased GST activity in progressive NAFLD samples was accompanied by a reduced pool of GSH, highlighting the depleted ability to combat oxidative stress, a causal factor for NASH (Hardwick et al., 2010). The antioxidant, *S*-adenosyl-l-methionine (SAM) provides the cysteine moiety for the generation of GSH. Several rodent studies have indicated a decrease in SAM on a high-fat diet (Kwon do et al., 2009; Buechler and Weiss, 2011). Furthermore, GSTs have a lower expression in Caucasians compared to African–Americans, who have a lower prevalence of NAFLD (Stepanova et al., 2010). The *GSTM1*-null genotype, shown to confer a higher risk of T2D, is also present at a higher frequency in NAFLD subjects compared to control (Hori et al., 2007, 2009). Thus, decreased activity of GSTs play a plausible role in NAFLD progression as a result of increased damage by oxidative stress (Table 3).

**Table 3:** **Phase II drug metabolizing enzymes and transporters implicated in the pathogenesis of NAFLD**.

Phase II DME/transporter	DRUGS metabolized/transported	Association with NAFLD
Glutathione-*S*-transferases (GSTs)	Chlorambucil (anti-cancer) Busulfan (anti-cancer)	GSTM2, GSTM4, and GSTM5 mRNA levels decreased in patients with steatosis and NASH (Ip et al., 2003)
	Cyclophosphamide (anti-cancer)	Overall GST activity decreased with disease progression, accompanied by a reduced pool of glutathione, highlighting the depleted ability to combat oxidative stress in NAFLD patients (Younossi et al., 2005)
		Lower expression in Caucasians compared to African–Americans (Hardwick et al., 2010)
		*GSTM1*-null genotype present at a higher frequency in NAFLD subjects (Stepanova et al., 2010)
Sulfotransferases (SULTs)	Acetaminophen (analgesic) Albuterol (β_2_-adrenergic agonist) Terbutaline (β_2_-adrenergic agonist)	SULT2B1b has anti-lipogenic properties by suppressing the LXR-SREBP1c interaction, resulting in decreased hepatic and serum level of lipids in *Ldlr*-null mice on a HFD (Hori et al., 2007, 2009)
	Hormonal contraceptives	*SULT1A2* expression is downregulated in NASH patients compared to control obese individuals (Bai et al., 2012)
		SULT1C4 and SULT4A1 have increased mRNA and protein levels in human NASH samples compared to control and steatosis samples (Younossi et al., 2005)
UDP glucuronosyltransferases	Non-steroidal anti-inflammatory drugs Opioids	Mice with severe hepatic steatosis induced by a high-fat and high-sucrose diet, display increased expression of *Ugt1a1* and *Ugt1a6* via interaction with CAR and PXR (Hardwick et al., 2012a)
	Anti-depressants Anti-psychotics	The UGT1A1*6 allele has a protective effect against NAFLD in a population of obese Taiwanese children (Osabe et al., 2008)
ABCC2	Pravastatin (statin) Vinblastine (anti-cancer)	Decreased in rodent models of obesity, NAFLD and NASH and normalized on roziglitazone treatment (Lin et al., 2009)
	Ceftriaxone (antibiotic)	rs17222723 and rs8187710 variants in the *ABCC2* are significantly associated with NAFLD patients and clinical and histological parameters (Geier et al., 2005; Fisher et al., 2009a; Martin et al., 2010)
Uptake transporters (NTCP, OATP1a1, 1a4, 1b2, 2b1, OAT2, and OAT3)	Atorvastatin (statin) Pravastatin (statin)	Downregulation of uptake transporters in the transition from steatosis to NASH rather than between control and steatotic samples (Sookoian et al., 2009)
	Rosuvastatin (statin) Non-steroidal anti-inflammatory drugs Captopril (anti-hypertension)

Sulfotransferases are involved in sulfation of several endogenous steroids and xenobiotics. The sulfation of oxysterols by SULT2B1b has anti-lipogenic properties by suppressing the LXR-SREBP-1c interaction, resulting in significantly lower hepatic and serum lipids as observed in low-density lipoprotein receptor (*Ldlr*)-null mice on a high-fat diet (Bai et al., 2012). Moreover, *SULT1A2* gene expression is downregulated in NASH patients compared to control obese individuals (Younossi et al., 2005). SULT2A1 is upregulated by PPARα agonists in primary human hepatocytes, but not in rat hepatocytes, due to the presence of a PPRE in the 5′ region of the gene (Fang et al., 2005; Runge-Morris and Kocarek, 2009). Thus, downregulation of PPARα observed in NAFLD may have implications in the altered expression of SULT2A1. However, only two SULT isoforms, SULT1C4 and SULT4A1, whose regulation and function are largely unknown, have increased mRNA and protein levels in human NASH samples compared to control and steatosis samples (Hardwick et al., 2012a). A previous association of SULT4A1 in deregulated metabolic homeostasis makes it a good candidate for further studies in the context of NAFLD (Kiba et al., 2009).

UDP glucuronosyltransferases are involved in the glucuronidation of 40–70% of all clinical drugs in humans (Wells et al., 2004). *UGT1A1*, *1A3*, *1A4*, *1A6*, and *2B4* are induced by PPARα-agonists in primary human hepatocytes and PPREs have been identified in these genes (Runge-Morris and Kocarek, 2009). Mice on high-fat and high-sucrose diet, which develop severe hepatic steatosis, display elevated expression of *Ugt1a1* and *Ugt1a6* mediated by CAR and PXR (Osabe et al., 2008). A study in a pediatric NAFLD population identified *UGT1A1* as a risk factor for NAFLD. The UGT1A1*6 allele in the coding region has a protective effect against NAFLD in obese Taiwanese children (Lin et al., 2009). UGT1A1 is involved in the glucuronidation of heme after breakdown to bilirubin. The ability of bilirubin to oxidize ROS may provide protection against the progression of NAFLD. Additionally, the high prevalence of unconjugated hyperbilirubinemia was detected in NAFLD patients (25.4%) that were diagnosed with less severe forms of NAFLD (Kumar et al., 2012). However, the absence of changes in glucuronidation activity in human steatosis and NASH liver samples warrants the need for further studies to investigate the role of UGTs in NAFLD (Hardwick et al., 2012a).

### Transporters

Solute carrier transporters are uptake transporters that transport molecules from the blood into the hepatocyte. Studies in rat and human samples have indicated a coordinated downregulation of uptake transporter genes in NASH, such as the sodium/bile acid transporter (*NTCP*), organic anion transporting polypeptide *1a1* (*OATP1a1*), *1a4*, *1b2*, *2b1*, *OAT2*, and *OAT3*. The expression of these transporters is significantly altered in the transition from steatosis to NASH rather than between control and steatotic samples (Fisher et al., 2009a; Lake et al., 2011). These changes appear to be hepatoprotective to prevent the accumulation of toxic intermediates and xenobiotics in the diseased liver. However, they have major implications in therapeutic regimens in NAFLD patients in terms of dose selection and side effects of drugs due to excessive accumulation (Table 3).

Transporters on the hepatocyte canalicular membranes are involved in the secretion of several endobiotics and xenobiotics *via* the bile. ABC-transporters are the most extensively studied and are altered in steatotic and NASH livers (Buechler and Weiss, 2011). Of particular interest is Mrp2/Abcc2, which is decreased in several rodent models of obesity, NAFLD and NASH and is normalized upon rosiglitazone treatment (Geier et al., 2005; Fisher et al., 2009a; Martin et al., 2010). Furthermore, the *rs17222723* and *rs8187710* variants in *ABCC2* significantly associate with NAFLD and clinical and histological parameters (Sookoian et al., 2009). Decreased levels of ABCC2 protein may result in hampered secretion of bile, leading to the accumulation of cholesterol and drug-related toxicities. As mentioned previously, this may result from impaired NRF2 function.

## Conclusion and Future Directions

The high prevalence of NAFLD is concerning in terms of general population health and also drug treatment regimens. A recent study in mice has identified that the feed-forward cycle of continuous exposure to high-fat diet over two generations leads to a significantly higher degree of obesity, NAFLD, insulin and leptin resistance, and epigenetic modifications resulting in increased lipogenesis and ER stress in future generations (Li et al., 2011). If these observations are also true for humans, the rising epidemics of obesity and NAFLD will expand exponentially in the absence of serious efforts to tackle these conditions.

With the widespread prevalence of NAFLD, the proportion of patients with steatotic livers undergoing drug therapies for various disorders has also increased. The variability of drug treatment responses in these patients highlights the need for personalized therapeutic regimens. As detailed in this review, several components of the drug metabolism pathway are significantly affected in the presence of NAFLD. Similarly, genetic variations in DMEs and nuclear receptors associate with NAFLD with either positive or negative prognosis. Hence, inter-dependent interactions and common confounding factors exist between the pathogenesis of NAFLD and altered drug metabolism. As a majority of the DMEs are also involved in the metabolism of steroids and other lipids, polymorphisms in DMEs resulting in non-functional proteins may further aggravate the prognosis of NAFLD. The utility of identified genetic associations to determine NAFLD disease susceptibility, improve drug sensitivity or prevent adverse drug reactions holds great potential. Further efforts to characterize DMEs and identify risk factors for adverse drug reactions or treatment efficacies in NAFLD populations may lead to the utilization of innovative interdisciplinary strategies to provide a better insight into the pharmacokinetic profile of drugs and their efficacy. Although the implementation of these findings in the clinic is still a long-term goal with hurdles to pass, novel technologies and increasing interest in this field continues to increase our understanding of NAFLD and its interactions with drug metabolism.

## Conflict of Interest Statement

The authors declare that the research was conducted in the absence of any commercial or financial relationships that could be construed as a potential conflict of interest.
